# Continuous monitoring using thermography can capture the heat oscillations maintaining body temperature in neonates

**DOI:** 10.1038/s41598-024-60718-y

**Published:** 2024-05-07

**Authors:** Aya Morimoto, Shinji Nakamura, Kosuke Koyano, Sae Nishisho, Yasuhiro Nakao, Makoto Arioka, Kota Inoue, Eri Inoue, Katsufumi Nishioka, Hirosuke Morita, Yukihiko Konishi, Konomu Hirao, Takashi Kusaka

**Affiliations:** 1 Department of Pediatrics, Faculty of Medicine, Kagawa University, Kitagun, Japan; 2 Maternal Perinatal Center, Faculty of Medicine, Kagawa University, Kitagun, Japan; 3 Creotech Ltd., Takatsuki, Osaka Japan

**Keywords:** Neonatology, Whole body imaging, Homeostasis

## Abstract

The body temperature of infants at equilibrium with their surroundings is balanced between heat production from metabolism and the transfer of heat to the environment. Total heat production is related to body size, which is closely related to metabolic rate and oxygen consumption. Body temperature control is a crucial aspect of neonatal medicine but we have often struggled with temperature measures. Contactless infrared thermography (IRT) is useful for vulnerable neonates and may be able to assess their spontaneous thermal metabolism. The present study focused on heat oscillations and their cause. IRT was used to measure the skin temperature every 15 s of neonates in an incubator. We analyzed the thermal data of 27 neonates (32 measurements), calculated the average temperature within specified regions, and extracted two frequency components—Components A and B—using the Savitzky–Golay method. Furthermore, we derived an equation describing the cycle—named cycle *T*—for maintaining body temperature according to body weight. A positive correlation was observed between cycle *T* and Component B (median [IQR]: 368 [300–506] s). This study sheds light on the physiological thermoregulatory function of newborns and will lead to improved temperature management methods for newborns, particularly premature, low-birth-weight infants.

## Introduction

Temperature control is of paramount importance for neonates after birth. If the body temperature is not properly controlled at 36.5–37.5 °C, respiratory disease, sepsis, and hypoglycemia can develop, particularly in preterm low-birth-weight infants (LBWIs), and these conditions are associated with an increased likelihood of developing intraventricular hemorrhage^1^. Heat loss by radiation, convection, conduction, and evaporation all occur via the body’s surface that is in contact with the surrounding environment, and this heat loss increases with the surface area. In newborn neonates with small bodies, the surface area per unit volume is larger than in adults and they have less subcutaneous fat as insulation and less stratum corneum, meaning that the heat loss is greater. On the other hand, preterm and small-for-gestational-age infants increase their metabolic rate with cooling, and their response is not typically much lower than that of term infants within 1 week after birth^2^. Normally, when body temperature decreases, the sympathetic nervous system is activated, peripheral vasoconstriction occurs, subcutaneous blood flow is reduced, and heat release is inhibited^3^. However, infants, particularly extremely LBWIs, do not exhibit peripheral vasoconstriction during the first 12 h of life, even if ambient temperatures are low^4^. Thus, we must consider the neonate's unique thermoregulation in systemic management.

In the neonatal intensive care unit (NICU), we often struggle to set incubator temperatures in response to temperature fluctuations. Therefore, in this study, we focused on contactless infrared thermography (IRT), which can continuously measure and visualize the radiant heat emitted from the whole-body skin surface. Since the beginning of the 1970s, researchers have used thermography to map the heat distribution of the skin^5–9^. Clark et al. reported research on neonates using IRT in 1980^9^, and Knobel-Dail et al. determined that the whole-body temperatures of extremely premature infants in an incubator can be captured using IRT^8^. In 2022, Hamada et al. established a new calibration method for IRT in an incubator and proved its accuracy and reliability^10^. Brown adipose tissue plays an important role in thermogenesis^11–13^ and Garcia-Beltran et al. used IRT to estimate brown adipose tissue activity at age 12 months^14^. As a tool for clinical diagnosis, Knobel-Dail et al. reported that the abdominal skin temperature was significantly lower in infants with necrotizing enterocolitis than in those without it^15^. Furthermore, some reports have indicated that IRT could be used for respiratory monitoring in infants^16,17^. Heimann et al. found significant differences in the temperature distribution of the whole body in the order (high > low): upper abdomen > back > arm > leg > head^18^.

Several previous reports found temperature fluctuations^9,18,19^, but their cause was unclear. A circadian rhythm of around 24 h and ultradian rhythm of around 90 min have been reported for the skin^20^. However, there has been no report of even shorter fluctuations or oscillation cycles in neonates. This is because it is impossible to continuously measure the stable body temperature of neonates, particularly LBWIs, in the clinical setting.

Metabolic processes that provide energy for the maintenance of homeostasis are closely linked to heat production. According to Kleiber’s law, the correlation between the logarithm of body weight and the logarithm of the basal metabolic rate (oxygen consumption) is close and has a slope of 0.75 for adult animals of various sizes. This means that the metabolic rate per kilogram^3/4^ of at-rest animals of different sizes, including adult humans, is independent of body size. Age, sex, body shape, and body composition affect metabolic rate. The age dependency of the metabolic rate makes it impossible to predict the metabolic rates of neonates and adults from an individual species with a single exponential function of weight, even if a higher exponent than Kleiber’s is used^2^. For the above reasons, we measured this fluctuation in human neonates, specifically LBWIs, to verify its cause. Our primary aim was to identify the relationship between the surface body area and heat fluctuation in neonates. The body temperature maintenance cycle of neonates is believed to be shorter than that of adults, and we hypothesized that the body surface area per unit volume would be related to the heat oscillation cycle, particularly in LBWIs. Accordingly, we derived a model equation to calculate the temperature maintenance cycle using the measurable body weight. The validity of this equation was evaluated by comparing it with the cycle extracted from the measured thermal images.

## Results

Between December 2018 and June 2021, 36 neonates underwent IRT measurements (49 in total) at Kagawa University Hospital. We excluded 6 neonates (7 measurements) because we were unable to measure continuous data for more than 25 min. In addition, 2 neonates (9 measurements) had abdominal surgery and 1 neonate (1 measurement) had suspected circulatory failure. Thus, we excluded a total of 9 neonates (17 measurements) due to a possible effect on the skin blood circulation. This left 27 neonates (32 measurements) for this study, whose characteristics are summarized in Table 1.

**Table 1:** Patient characteristics.

Neonates (n = 27, 32 measurements)	Median [IQR]
Birth weight (g)	1092 [860–1936]
Gestational age (weeks)	28.3 [27.1–34.3]
Male, n (%)	12 (44)
Postnatal age (days)	20 [8–47]
Weight at measurement (g)	1430 [1029–1896]

Clinical data on 32 measurements from 27 neonates.

Data are presented as n (%) or median [IQR].

IQR, interquartile range.

**Figure 1:**
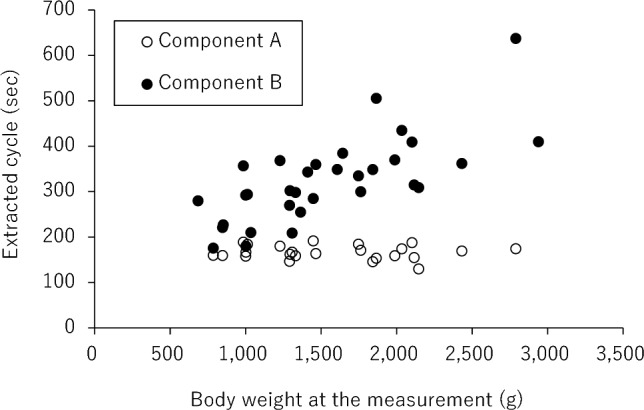
Component A (white dot) and Component B (black dot) were extracted with the Savitzky–Golay method from 32 measurements.

**Figure 2:**
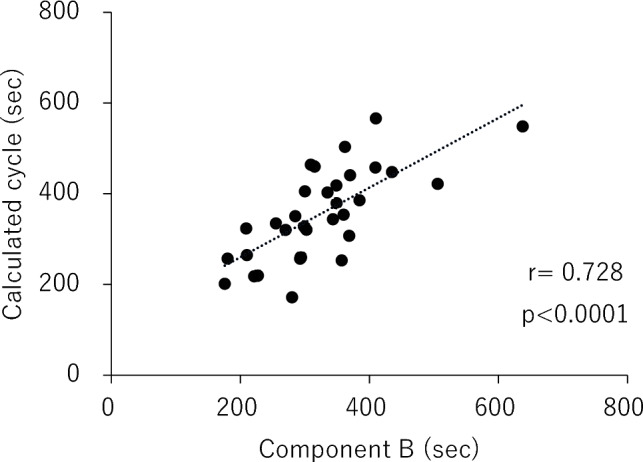
The cycle calculated using Eq. (5) (cycle *T*) was correlated with Component B, which was extracted using the Savitzky–Golay method. Spearman’s rank correlation coefficient was used for nonparametric data.

In this study, the temperature inside the incubator (median [interquartile range (IQR)]: 28.7 °C [28.3–30.5 °C]) and humidity (median [IQR]: 53% [50%–63%]) were stable (Table 2). Nine neonates received CPAP while 7 received mechanical ventilation, which might have affected the temperature in the incubator. We analyzed the sequential thermal data (median measurement time [IQR]: 48 [37–68] min) and extracted Component A and Component B from the time-series surface temperature using the Savitzky–Golay method. The results showed that Component A remained nearly constant, even at different body weights (median [IQR]: 171 [158–192] s). Component B (median [IQR]: 368 [300–506] s) was correlated with body weight at measurement (Fig. 1). Furthermore, using α = 0.13, β = 1, γ = 45, Xt = 0, and Yt =  − 10.2, cycle* T* was calculated from the weight at measurement and Eq. (5). A positive correlation was observed between cycle* T* and Component B with Spearman’s rank correlation coefficient (r = 0.728, *P* < 0.0001) (Fig. 2).

**Table 2:** Factors affecting temperature in incubator and analysis area for 32 neonates.

No.	Temperature inside an incubator (°C)	Humidity (%)	Medical equipment	Analysis area	Analyzed the number of pixels
1	28.4	64	CPAP	Abdomen	144
2	28.0	67	CPAP	Abdomen	256
3	33.1	65	MV	Abdomen	289
4	32.9	55	MV	Whole body	15,928
5	28.2	48	–	Whole body	19,206
6	30.2	51	–	Whole abdomen	3430
7	28.4	54	–	whole body	9126
8	33.5	55	CPAP	Abdomen	320
9	27.1	50	–	Abdomen	1196
10	32.6	57	MV	Abdomen	1681
11	29.5	50	MV	Abdomen	651
12	28.7	64	CPAP	Abdomen	676
13	28.3	66	CPAP	Abdomen	961
14	27.3	66	CPAP	Abdomen	1216
15	33.0	60	MV	Abdomen	567
16	28.7	50	–	Abdomen	285
17	30.8	52	–	Abdomen	550
18	30.2	50	–	Abdomen	2340
19	29.7	51	–	Abdomen	460
20	27.7	53	–	Abdomen	660
21	28.3	49	–	Back	252
22	30.4	50	–	Abdomen	154
23	28.3	50	MV	Abdomen	144
24	26.9	76	CPAP	Abdomen	144
25	27.4	74	CPAP	Abdomen	143
26	29.0	51	–	Abdomen	150
27	32.8	50	MV	Abdomen	289
28	30.7	55	–	Abdomen	324
29	28.7	63	CPAP	Abdomen	132
30	28.6	52	–	Abdomen	81
31	28.7	51	–	Abdomen	120
32	29.6	50	–	Abdomen	120

Clinical data on 32 measurements from 27 neonates.

CPAP, continuous positive airway pressure; MV, mechanical ventilation.

**Figure 3:**
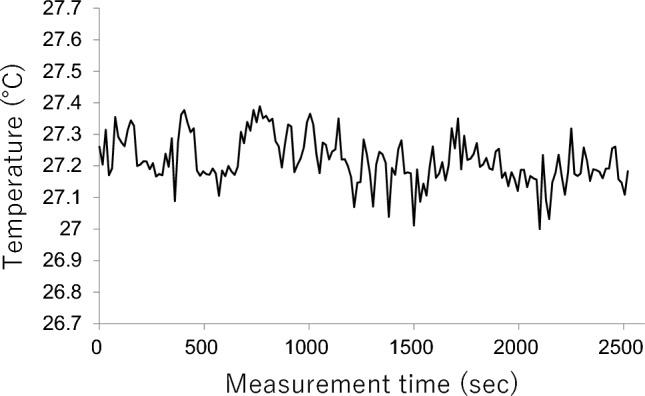
Temperature measurement data from a vacant incubator. A static thermal image was obtained every 15 s, the average temperature within specified regions was calculated, and a time-series temperature graph was plotted.

Moreover, we examined the effects of thermal wind. We set the incubator temperature to 28.0 °C and the humidity to 60%. We measured a vacant incubator with IRT for 40 min and measured the heat radiation from a mattress usually used for a neonate. The temperature was stable at around 27 °C (Fig. 3).

## Discussion

Two frequency components were extracted from the sequential thermal data with IRT. Component A was constant regardless of body weight, whereas Component B was correlated with body weight at the time of measurement. In addition, we identified an equation that leads to a thermoregulatory cycle (Component B) that varies with body weight. These components may enable temperature control based on individual thermoregulatory capacities. No previous study has examined the heat cycle every few minutes in neonates. We considered that the body surface area per unit volume is related to the heat oscillation cycle, particularly in LBWIs, and therefore derived a model equation to calculate the temperature maintenance cycle, using the measurable body weight. This is the first study to report that the heat oscillation cycle varies with body weight.

First, there are several relevant reports in the literature concerning entities similar to Components A and B in terms of frequency length. In 1983, Gordon et al. reported that the neural and motor components, including temperature regulation, include a variety of rhythmic processes that cover a frequency range of at least five orders of magnitude. The frequency range of the thermo-afferent system (peripheral thermoreceptors and ascending connectives in the brain and spinal cord) is relatively small (1–7 Hz), whereas the neural integrative and motor divisions exhibit oscillating activity ranging from 10^−4^ to 10 Hz^21^. In particular, the human neural control of body temperature-related evaporative water loss (0.008 Hz) identified in their study is equivalent to the frequencies of Components A and B.

In a recent study, Qiao et al. reported the development of novel fluorescent polymer probes for accurate and simultaneous measurements of intracellular temperature and ATP during oxidative phosphorylation. They found that ATP fluctuated for 2 min during oxidative phosphorylation^22^. Yamanaka et al. reported that cellular thermogenesis compensates for environmental temperature fluctuations to maintain intracellular temperature, although they did not mention the cycle length^23^. We assume that such fluctuations at the cellular level or in nervous activity may reflect Components A and B, considering the lengths of the cycles. Component A was constant regardless of body weight, and the temperature was stable in a vacant incubator (Fig. 3). We speculate that Component A could be caused by a temperature-control system in an incubator or the functional influence of IRT.

Next, because heart rate (HR) depends on body weight or postnatal age, we hypothesize that Component B reflects the circulation, particularly cutaneous blood flow. Iriki et al. reported spontaneous thermoregulatory oscillations in cutaneous efferent sympathetic activity^24^. The observed changes in ear skin temperature in rabbits are indicative of oscillations in blood flow, and the spontaneous nature of these events is characteristic of the activity of thermoregulatory mechanisms when an animal is in a thermoneutral zone. Lossius et al. reported a connection between skin arteriovenous anastomosis (AVA) flow fluctuations and HR variability in infants. They found that individual AVA constrictions were accompanied by a diphasic HR response, indicating the presence of an autonomic rhythm that affected both skin AVA activity and HR variability^25^. Therefore, Component B, which correlates with body weight, is presumed to comprise skin blood flow controlled by autonomic rhythms. The blood supplying systemic organs plays a role in heat transportation, and heat transported by convection in the vessel is released to the skin^26^. Skin temperature typically reflects cutaneous blood flow^20^. Because the vascular plexus in the dermis develops after birth^27^, IRT can capture minute oscillations of skin blood flow in neonates, who have less fat as a heat insulator.

The equations that we developed indicate that the body temperature maintenance cycle *T* is 45 s for a body weight of 500 g, 234 s for a body weight of 1,000 g, and 473 s for a body weight of 2000 g, and that the body temperature changes by 0.1–0.2 °C in these cycles. That means that neonates have the appropriate ambient temperature if they have around a 4-min thermal cycle for a 1000 g body weight. On the other hand, if the measured thermal cycle deviates from the cycle derived from the equation, we should consider that the patient is not in the appropriate temperature environment or that there is a serious general condition, such as necrotizing enterocolitis or systemic infection, that may cause circulatory failure. The assessment of skin blood flow or circulatory blood flow might be a new method for diagnosing and monitoring circulatory failure.

Homeothermy requires a balance among heat production, skin blood flow, sweating, and respiration in such a way that changes in heat loss or gain from the environment are precisely compensated^2^. Moreover, human skin blood flow can range from almost zero (in conditions of whole-body and/or local cooling) to up to 60% of cardiac output in conditions of severe heat stress^28^. IRT could be a new way for circulatory assessment when the skin is considered an organ.

However, there is some limitation to the IRT evaluation of the circulatory conditions underlying changes in body temperature. Hirata et al. reported two factors by which the skin temperature is determined: blood temperature and skin blood flow. Venous temperature is lower than that of arterial blood and the peripheral limb temperature is lower than that of the trunk. Moreover, they reported that the skin blood flow does not change, even with a skin temperature increase^29^. In this situation, it is impossible to predict the skin blood flow based on skin temperature. Therefore, we need to evaluate not only IRT, but also consider other vital signs.

In the analysis, thermal data during routine care were excluded and the pre- and post-analysis data were connected for two neonates. We did not have an acclimatization period, which is generally about 10–15 min. Because data were recorded once every 15 s, we excluded any data that showed the nurse’s hand during routine care. Therefore, in terms of the data, there was little influence of the nurse’s hands. The temperature fluctuated only temporarily and did not affect the cycle, although the basal part of the cycle seemed to fluctuate. We believe the acclimatization period should be taken into account and will consider how to handle this matter in a future study. Moreover, we analyzed all neonates together, regardless of whether or not they used CPAP or mechanical ventilation, which might have affected the temperature inside the incubator. When we are better able to elucidate neonatal thermoregulation, we will need to distinguish between those effects and analyze them accordingly.

The incubator was constructed of 5-mm-thick polymethyl methacrylate (PMMA), which absorbs infrared radiation from the neonates. PMMA has C-O stretching vibration absorption as well as C–H, CH_3_, and C–OH angular vibration absorption in the T450SCA measurement range (7.5–13 µm). It acts as a light absorber, so no significant reflection occurs. This means that PMMA does not reflect infrared radiation from the neonates. We believe that even if there are mild reflections from the PMMA, it does not affect the heat oscillations of neonates with a temperature of 34 °C or higher. In addition, we do not believe that a temperature of around 27 °C in an incubator affects the heat oscillations of neonates with a temperature of 34 °C or higher. Such as change would affect the rate of heat dissipation, which might influence the thermal cycle. However, a change in the surrounding temperature of up to 1 °C in a neutral temperature environment would not have a substantial impact. We consider the variation in the periodicity (distribution of the periodicity) to be enough to absorb the error.

In this study, we found that the heat cycle was regular, but we were unable to identify the factors controlling Component B. We believe that this heat cycle is an important factor in the maintenance of temperature homeostasis, especially in neonates, because they have a large surface body area per body weight. We speculate that this cycle might be one of the mechanisms limiting viability. By building on the present results, we hope to find another factor that controls thermoregulation. Therefore, we should simultaneously measure temperature, HR, and blood pressure because circulatory assessment methods are completely different from conventional management methods using core body temperature as an index. IRT is a stress-free measurement method for vulnerable neonates, and the discovery of temperature maintenance cycles could be an innovative method for temperature management in the NICU.

## Conclusion

In this study, we identified two kinds of temperature cycles using IRT. The cycle to maintain body temperature was correlated with body weight, and we derived an equation explaining the maintenance of body temperature with body weight. Continuous monitoring of the skin temperature using IRT has the potential to be a novel way to manage body temperature.

## Materials and Methods

This prospective observational study was performed at Kagawa University Hospital. The subjects were hospitalized in the NICU between December 2018 and June 2021. Room temperature was standardized at around 25 °C and humidity was around 50%. However, external factors such as variations in environmental temperature and variable air velocity and humidity can influence accurate skin measurement with IRT^30^. Because oxygen consumption is lowest in a neutral thermal environment, all neonates in this study were measured in a closed incubator. We recorded the usage of CPAP and mechanical ventilation, which might have affected the temperature in the incubator.

### Ethical approval and informed consent

This study was approved by the Regional Committee on Biomedical Research Ethics of Kagawa University (approval number: H29-042) and conducted in accordance with the Declaration of Helsinki. We confirmed that all methods were performed in accordance with relevant guidelines and regulations. The parents of all neonates enrolled in this study provided written informed consent after receiving a full explanation of the research. Because neonates requiring hospitalization in the NICU are vulnerable, we endeavored to shorten the measuring time for the analysis. In addition, we immediately stopped the measurement when the neonate was crying or showing any signs of distress.

### Setup of the thermography and measurement in the incubator

Before our experiment, we assessed the environmental temperature in three places of the incubator—the head and foot of infants and part of the sensor module on the ceiling—in an incubator with a platinum digital thermometer (Platinum Thermo SN-3400, Netsuken Co., Ltd., Tokyo) to verify the difference in temperature at each location in the incubator. All temperatures were stable and there were no differences among the three places.

**Figure 4:**
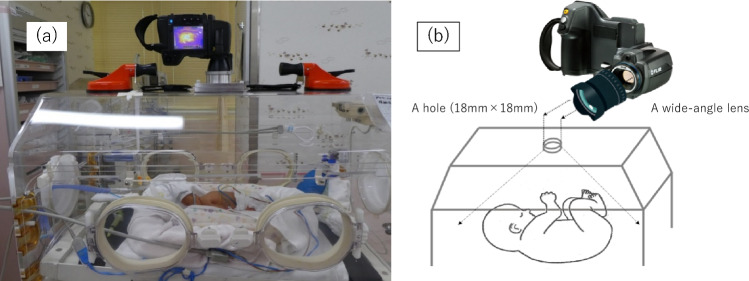
(**a**) Photograph showing the actual IRT setting. (**b**) The IRT device was placed on the ceiling of the incubator and took thermal images through the hole (18 mm × 18 mm).

We used ATOM Medical (Incu i®, Atom Medical Corporation, Tokyo) and Dräger (Isolette 8000® and Isolette 8000 plus®,  Drägerwerk AG & Co. KGaA, Germany) incubators. Each incubator was constructed of 5-mm-thick polymethyl methacrylate (PMMA), which absorbed some of the infrared radiation from the infant. Therefore, we fixed the camera in place with an elastic band and suction cup on the incubator and directly performed thermography imaging through a hole (18 mm × 18 mm) drilled in the ceiling of the incubator that had already been opened for measurement of the neonates’ body weight (Fig. 4a and b). For the IRT temperature and the ambient temperature in the incubator to equalize, we waited 15 min before starting the recording. We automatically collected a thermal static image every 15 s for about 1 h.

During the measurement, the necessary routine care was conducted by nurses, including administration of breast milk by nasogastric tube, diaper changing, and measurement of HR, blood pressure, respiratory rate, and axillary temperature.

### Infrared thermography

We used the infrared thermograph CPA-T450SCA (FLIR, Chino Corporation, Tokyo). IRT visualizes the temperature of an object’s surface as captured by a thermal camera. Infrared radiation is naturally emitted from all materials, and the radiation intensity depends on its temperature, emissivity, and wavelength. In infrared measurements, it is necessary to take into account the transmittance of infrared light in the atmosphere. There is a band in the atmosphere called the “window of the atmosphere” that transmits infrared radiation well. The spectral range of thermography is 7.5–13 µm, which corresponds to long-wave infrared and can transmit infrared radiation well. We used a lens made of germanium with an anti-reflective coating and a transmittance of > 98% at 8–12 µm. A wide-angle lens (90° lens CPZ-4090, T197412, Chino Corporation, Tokyo) was used instead of a standard lens to capture the neonates’ whole body. In this study, the object was measured with an emissivity of 0.95, a distance of 1.0 m, a reflected apparent temperature of 22.0 °C. The atmosphere's temperature was measured as 23.0 °C, with a relative humidity of 50% and a transmittance of 0.99. The temperature of the external optics (lens) was measured as 20.0 °C with a transmittance of 1.00. We calculated the field of view (FOV) from the diameter of a can containing warm water at about 40 °C and the number of pixels. The horizontal FOV was 640 mm, and the vertical FOV was 480 mm. The noise equivalent temperature difference was < 30 mK and the frame rate was 30 Hz.

### Method for obtaining temperature time-series data from thermal images

**Figure 5:**
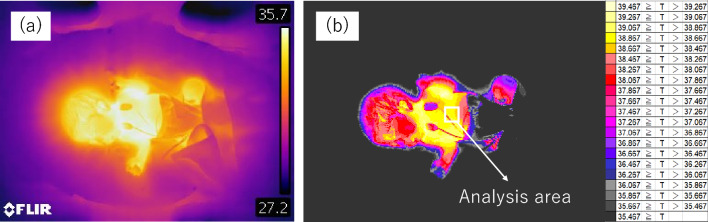
A representative example of a thermal image captured using IRT. (**a**) An IRT image of a neonate during the measurement. The IRT was placed on the ceiling of the incubator, which enabled visualization of the temperature distribution in the whole neonate body. (**b**) We created the gradient images at 0.2 °C intervals from a neonatal image using IRT and specified a target region of the thermal image visually.

Thermal images of each neonate were captured every 15 s by IRT. First, a gradient image was created from the neonatal thermal image with a range of 4 °C at 0.2 °C intervals. The gradient image was based on the maximum temperature and each 0.2 °C interval is shown in the same color (Fig. 5a and b).

**Figure 6:**
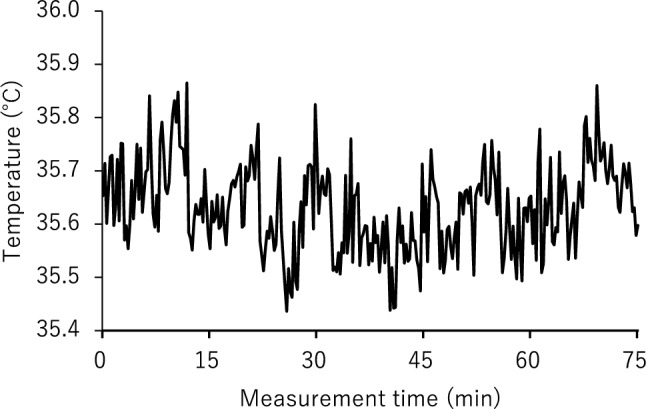
Representative example of measurement data from a neonate. A static thermal image was obtained every 15 s, the average temperature within specified regions was calculated, and a time-series temperature graph was plotted.

Next, a periodic temperature change was seen in the whole body and synchronized in the whole abdomen so that we could capture the same heat cycle in any area chosen (Supplementary Figure S1). We chose an analysis area that was not affected by the ECG cable or a staff member’s hand. Therefore, the target area and its size differed among measurements. The analysis area ranged in size from around 1 to 4 cm^2^. Each analysis was performed on data in which images of only the neonate were continuous for more than 25 min (more than 100 images). We showed the number of pixels for each piece of analyzed data (Table 2). Finally, we calculated the average temperature in the target area and converted it into time-series data (Fig. 6). The following data exclusion criteria for the analysis were applied: (1) when the analysis range fluctuated for the thermal image; (2) when more than 25 min of continuous data could not be obtained due to frequent overlapping of obstacles such as staff member’s hands in the analysis range; and (3) when the neonate started to cry during the filming.

### Frequency separation using the Savitzky–Golay method for the time-series data

We performed cyclic separation with the Savitzky–Golay method, used for data smoothing^31^. The time-series temperature data obtained here are denoted ${a}_{i}$.

1. Noise data extraction (applying Savitzky–Golay 9-point smoothing)

We performed smoothing of 9 discrete points of the measurement data.

$$\frac{-21\times {a}_{1}+14\times {a}_{2}+39\times {a}_{3}+54\times {a}_{4}+59\times {a}_{5}+54\times {a}_{6}+39\times {a}_{7}+14\times {a}_{8}-21\times {a}_{9}}{231}$$

**Figure 7:**
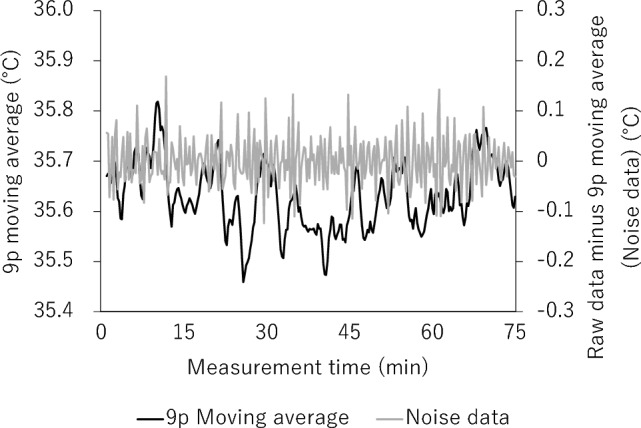
The 9p moving average (black; 9 discrete points) was plotted using measured data (Fig. 6) and the noise data were isolated (gray).

The smoothing corresponds to the time ${a}_{5}$ (hereafter denoted ${b}_{i}$). The first separation was performed with ${a}_{i}-{b}_{i}$ as the “noise data” (Fig. 7).

2. Extraction of Components A and B (applying Savitzky–Golay 25-point smoothing)

$$\left(-253\times {b}_{1}\right.-138\times {b}_{2}-33\times {b}_{3}+62\times {b}_{4}+147\times {b}_{5}+222\times {b}_{6}+287\times {b}_{7}+342\times {b}_{8}+387\times {b}_{9}+422\times {b}_{10}+447\times {b}_{11}+462\times {b}_{12}+467\times {b}_{13}+462\times {b}_{14}+447\times {b}_{15}+422\times {b}_{16}+387\times {b}_{17}+342\times {b}_{18}+287\times {b}_{19}+222\times {b}_{20}+147\times {b}_{21}+62\times {b}_{22}-33\times {b}_{23}-138\times {b}_{24}-253\times \left.{b}_{25}\right)/5175$$

**Figure 8:**
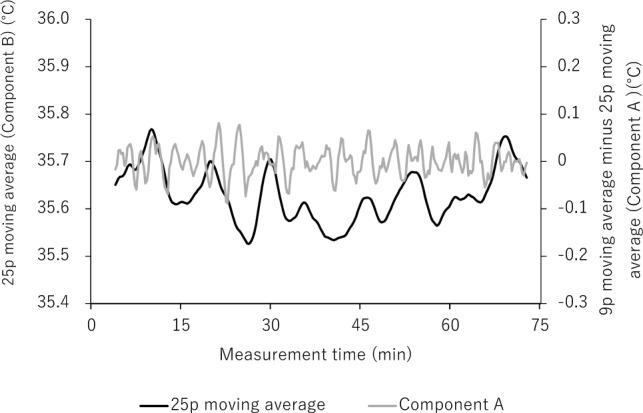
The smoothing of 25 discrete points was performed on the smoothed data of 9 discrete points (Fig. 7) and the data were named “Component A” (i.e., after the smoothed data of 25 discrete points were subtracted from the smoothed data of 9 discrete points). The data after the smoothing of the 25 discrete points were called “Component B”.

In the above equation, the data after smoothing corresponds to the time of b_13_ (hereafter denoted ${c}_{i}$). The 25-point smoothed data ${c}_{i}$ are named Component B and comprise the longest period of data among the separated data (Fig. 8). In addition, ${b}_{i}-{c}_{i}$ is named Component A. The peaks in each cycle of Components A and B were detected and averaged.

We compared the results extracted by Fourier transform and the Savitzky–Golay method. The data obtained using Fourier transform were inaccurate due to unclear periodic peaks, indicating the need for more long-term data in neonates for Fourier transform. Components A and B comprise the original measurement data, including several noise data sets due to the neonates’ body movement, and they effectively explain the characteristics of the data. The Savitzky–Golay method functions as a high-pass filter or low-pass filter when the noise data are separated. When Components A and B are added together, the data are exactly the same as the original measurement data. This means that no data were excluded, and this was visually confirmed.

For these reasons, we used the Savitzky–Golay method to extract the necessary periodic components from the temperature time-series data.

### Derivation and evaluation of the periodic simulation equation from the surface area/volume

We focused on the thermal cycle of Component B obtained by the Savitzky–Golay method. The temperature measured by IRT is the heat loss by radiation from the body’s surface. The larger the body surface area, the greater the heat release. Volume (body weight) is related to heat capacity; the smaller the body weight, the easier it is to lower the body temperature. Furthermore, if thermostatic animals are similar in shape, the body surface area can be determined from the body weight, and we surmised that it would be possible to determine the pattern of heat production and loss, which would form a cycle, using body weight as a variable.

Initially, we wondered if it would be possible to approximate the neonate using a three-dimensional cylinder. To determine the volume and surface area of a cylindrical shape, it is necessary to determine two variables: the radius of the circle and the length of the cylindrical part. This is possible if there is a certain relationship between the radius and length but it is currently difficult to perform measurements to obtain such a relationship in neonates. Therefore, we decided to use the simplest sphere. From the viewpoint of heat loss, we disregarded the limbs with the largest surface area, which would have higher heat loss efficiency, and replaced them with α, as described below. The units are expressed in the centimeter-gram-second (CGS) system of units.

Surface area formula for a sphere

$$4\uppi {r}^{2}$$

Volume formula for a sphere:

$$\frac{4{\pi r}^{3}}{3}$$

Here, *r* is the radius of the sphere (cm).

Volume is largely proportional to the surface area, and the decrease in temperature with heat loss is inversely proportional to volume, which is known as heat capacity. Therefore, the temperature decrease with heat loss is expressed by the equation below.

When we divide the surface by volume, we can get the surface area per unit volume, which indicates a larger temperature drop with a larger value.

1 $$\mathrm{\alpha }\frac{4\pi {r}^{2}}{\left(\frac{4\pi {r}^{3}}{3}\right)}=\frac{3\alpha }{r}$$

Here, *α* is the influence coefficient of volume on the surface area; α is a proportional coefficient and is both unitless and dimensionless. Although the heat dissipation efficiency differs between the trunk and extremities, the ratio of the trunk to the extremities is the same as long as the body is analogous. Therefore, the difference between them is absorbed by the proportional coefficient α.

The cycle for maintaining body temperature is shorter when the temperature decreases quickly and vice versa. Therefore, the cycle for maintaining the temperature is the inverse of Eq. (1).

2 $$\frac{r}{3\mathrm{\alpha }}$$

Next, it is converted to body weight, which is the measuring variable, because it is impossible to measure *r* (cm)*.*

$${\text{W}}=\upbeta \frac{4\pi {r}^{3}}{3}$$

Here, *W* is body weight (g) and $\beta$ is the proportionality coefficient for exchanging the body weight, which is the density of the biological tissue (g/cm^3^).

3 $${\text{r}}=\sqrt[3]{\frac{3W}{4\pi \beta }}$$

Thus, we can convert the radius to the body weight.

We substitute *r* into Eq. (2)

4 $$\frac{r}{3\alpha }=\frac{1}{3\alpha }\sqrt[3]{\frac{3W}{4\pi \beta }}$$

Finally, because the position of the zero point is unknown, the moving variables *x*_*t*_ and *y*_*t*_ in the x-axis (the body weight) and y-axis (the temperature maintenance cycle *T*) directions and the magnification variable *γ* for the overall scaling are introduced to complete the simulation equation for the cycle *T*.

5 $$T=\upgamma \left(\frac{1}{3\alpha }\sqrt[3]{\frac{3\left(W+{x}_{t}\right)}{4\pi \beta }}+{y}_{t}\right)$$

Here, *T* is the cycle (s), *x*_*t*_ is the moving variables for body weight (g), *y*_*t*_ is the moving variables for the cycle (s), and *γ* is the magnification variable (s/cm).

The validity of Eq. (5) was evaluated by comparing cycle T, which is calculated from the weight at measurement time, and Component B, which is extracted using the Savitzky–Golay method.

### Data analysis

GraphPad Prism 10.0.1 (GraphPad Software, La Jolla, CA) was used for all statistical analyses. All values are presented as the median [IQR]. Spearman’s rank correlation coefficient was used for nonparametric data such as Component B and cycle *T*. A *p*-value < 0.05 was considered significant.

### Supplementary Information

Supplementary Information 1.

Supplementary Information 2.

## Data Availability

The datasets generated during and/or analyzed during the present study are available from the corresponding author on reasonable request.
