# Supplementary material for: Continuous monitoring using thermography can capture the heat oscillations maintaining body temperature in neonates
Source: Sci Rep. 2024 May 7;14:10449. doi: 10.1038/s41598-024-60718-y (PMC11076520; doi:10.1038/s41598-024-60718-y)
Supplement: Supplementary file 1 — Supplementary Information 1. [file 41598_2024_60718_MOESM1_ESM.docx]

**Supplementary information**

**Article in *Scientific Reports***

**Continuous monitoring using thermography can capture the heat oscillations maintaining body temperature in neonates**

Authors and affiliation

Aya Morimoto^1^, Shinji Nakamura^1^, Kosuke Koyano^2^, Sae Nishisho^1^, Yasuhiro Nakao^1^, Makoto Arioka^2^, Kota Inoue^1^, Eri Inoue^1^, Katsufumi Nishioka^1^, Hirosuke Morita^2^, Yukihiko Konishi^1^, Konomu Hirao^3^, Takashi Kusaka^1^*

^1^ Department of Pediatrics, Faculty of Medicine, Kagawa University, Kitagun, Japan

^2^ Maternal Perinatal Center, Faculty of Medicine, Kagawa University, Kitagun, Japan

^3^ Creotech Ltd., Takatsuki, Osaka, Japan

Supplementary figure legend

Supplementary Figure S1.

A thermal image obtained every 15 s from a neonate lying in a prone position. The heat fluctuation in the whole body is shown.
