# Supplementary figures and images for: Continuous monitoring using thermography can capture the heat oscillations maintaining body temperature in neonates
Source: Sci Rep. 2024 May 7;14:10449. doi: 10.1038/s41598-024-60718-y (PMC11076520; doi:10.1038/s41598-024-60718-y)

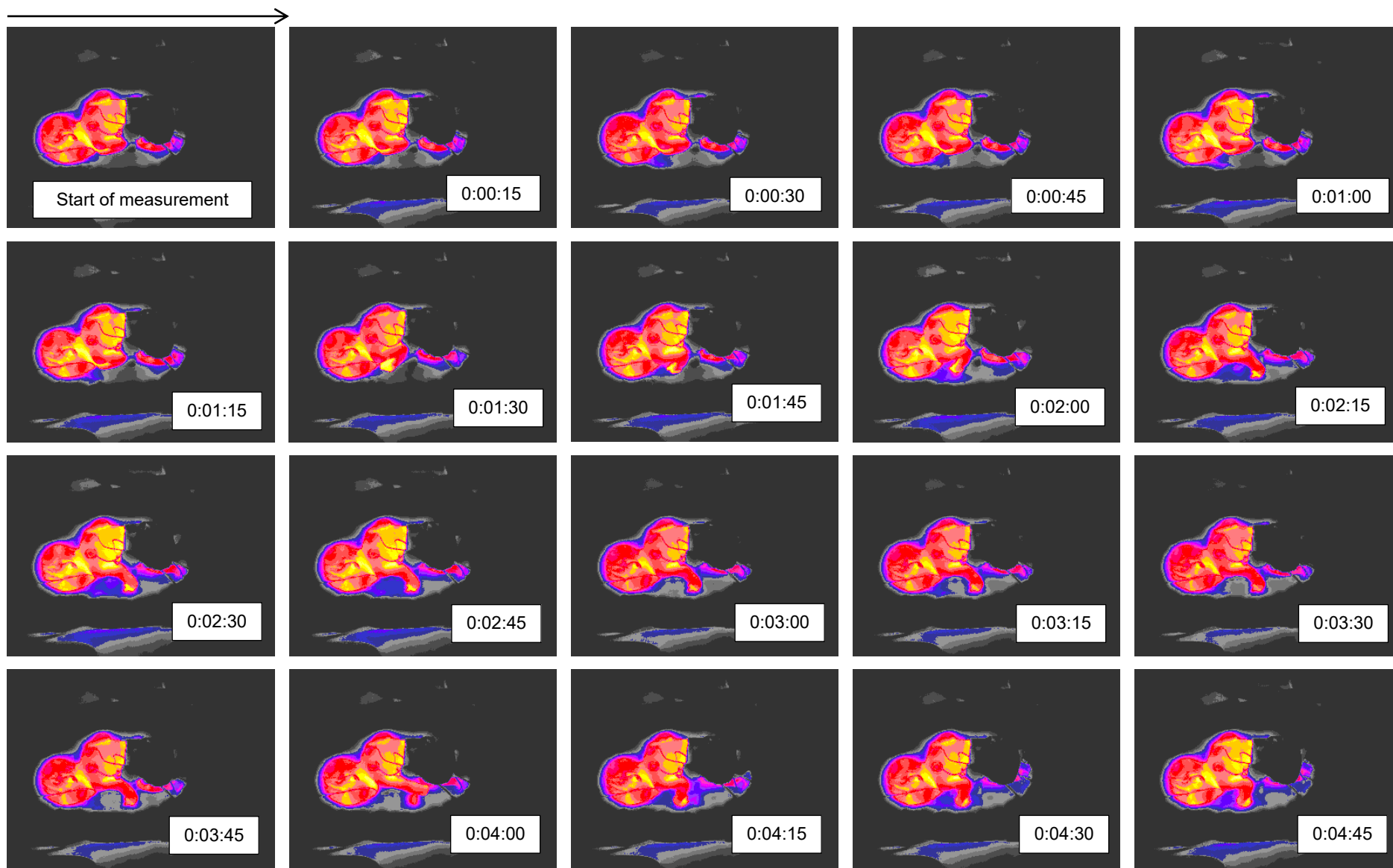

Supplementary Figure S1-A.

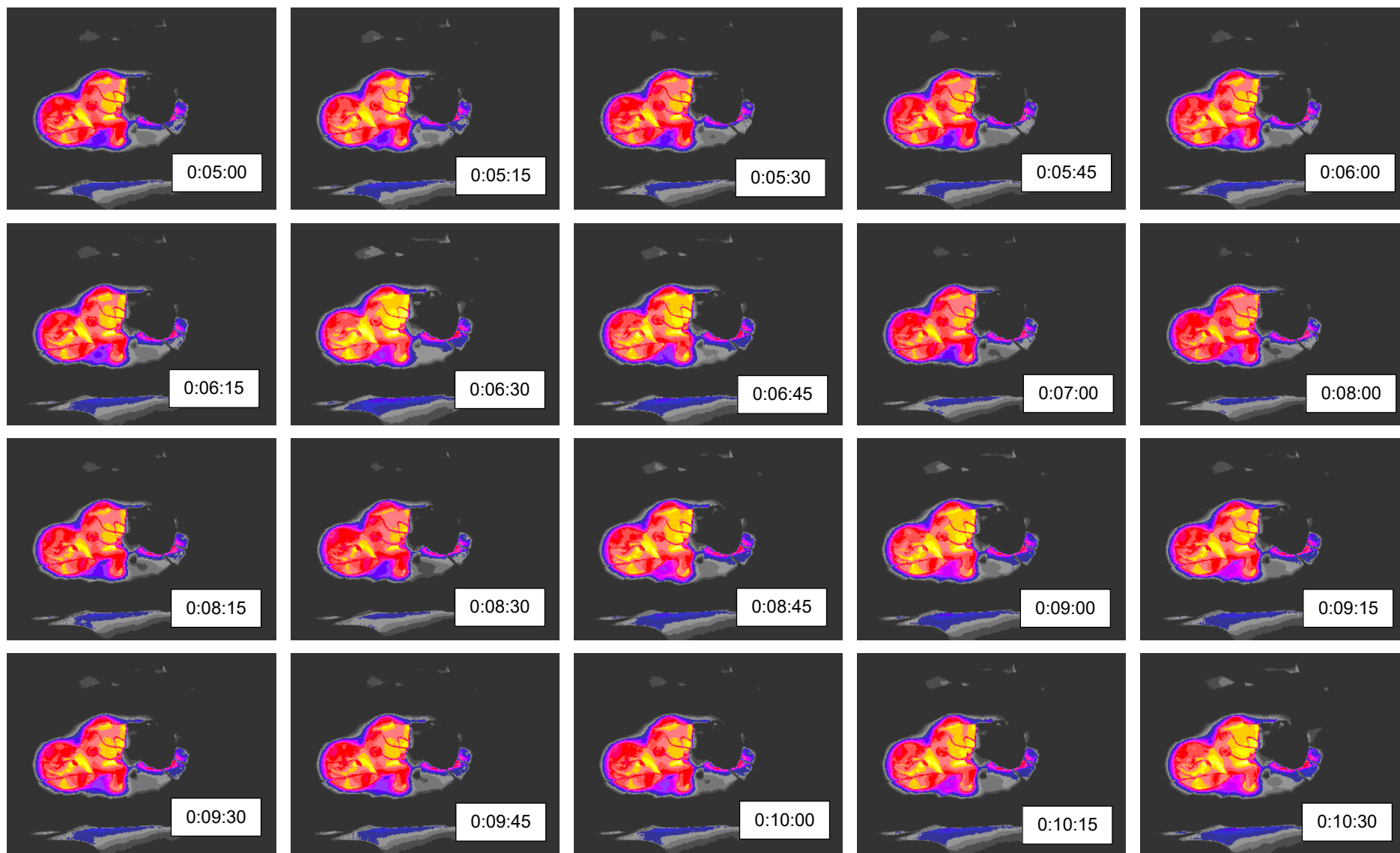

Supplementary Figure S1-B.

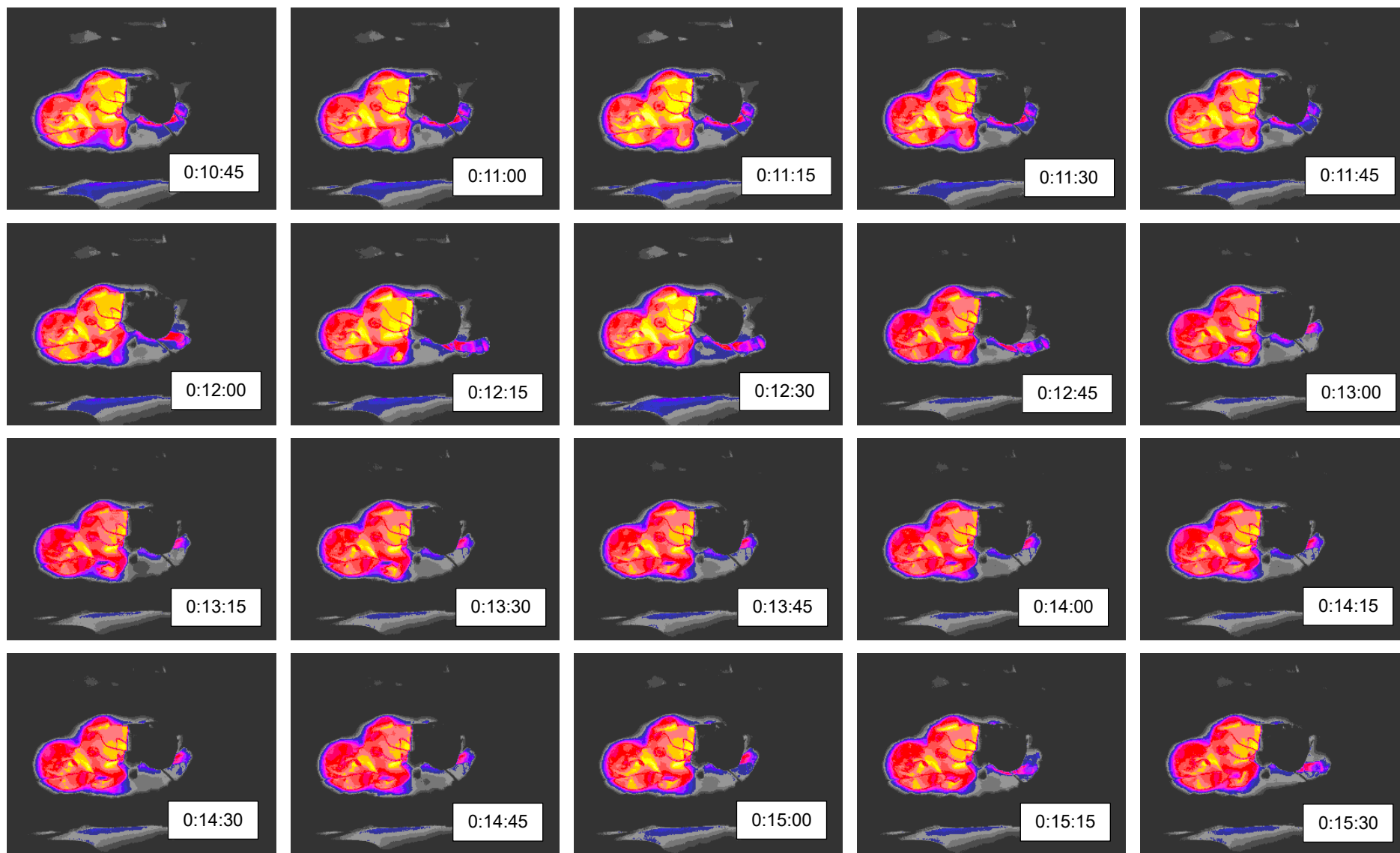

Supplementary Figure S1-C.

Supplement: Supplementary file 2 — Supplementary Information 2. [file 41598_2024_60718_MOESM2_ESM.pdf]
